# Development of a noninvasive and label‐free imaging system for human interfollicular epidermal stem cells based on cell morphology

**DOI:** 10.1111/srt.13887

**Published:** 2024-07-31

**Authors:** Katsuma Miyachi, Takeru Shiraishi, Ayumi Sanada, Yoshie Ishii, Osamu Hirose, Takaaki Yamada, Toshio Igarashi, Seiji Hasegawa, Masaru Arima, Yohei Iwata, Kazumitsu Sugiura, Hirohiko Akamatsu

**Affiliations:** ^1^ Research Laboratories Nippon MENARD Cosmetic Co., Ltd Nagoya Aichi Japan; ^2^ Department of Applied Cell and Regenerative Medicine Fujita Health University School of Medicine Toyoake Aichi Japan; ^3^ Department of Dermatology Fujita Health University School of Medicine Toyoake Aichi Japan; ^4^ Nagoya University‐MENARD Collaborative Research Chair Nagoya University Graduate School of Medicine Nagoya Aichi Japan

## 1 RESEARCH LETTER

Interfollicular epidermal stem cells (IFE‐SCs), on the basement membrane of the adult epidermis, contribute to tissue homeostasis and regeneration.^1^, ^2^ CD271, integrin beta 1, and melanoma‐associated chondroitin sulfate proteoglycan (MCSP) have been recognized as distinctive marker genes for IFE‐SCs.^3^, ^5^ Cultured IFE‐SCs can be used to produce three‐dimensional epidermal sheets for transplantation medicine. However, IFE‐SCs in the epidermis exist in limited quantities, with individual and regional variations. Ideally, skin should be preferentially collected from individuals or regions with abundant IFE‐SCs. However, quantifying IFE‐SCs at the collection site requires invasive and impractical methods, involving skin biopsy donors and analysis using antibodies for IFE‐SC markers. Therefore, novel approaches are required to analyze IFE‐SCs noninvasively. Although studies have explored noninvasive, in vivo imaging of IFE‐SCs, these investigations have been limited to mouse studies owing to the need of introducing fluorescent proteins and probes into cells.^6^ In vivo analysis of IFE‐SCs in humans has not been achieved to date. Therefore, this study aims to develop a noninvasive and label‐free method for analyzing human IFE‐SCs.

**FIGURE 1:**
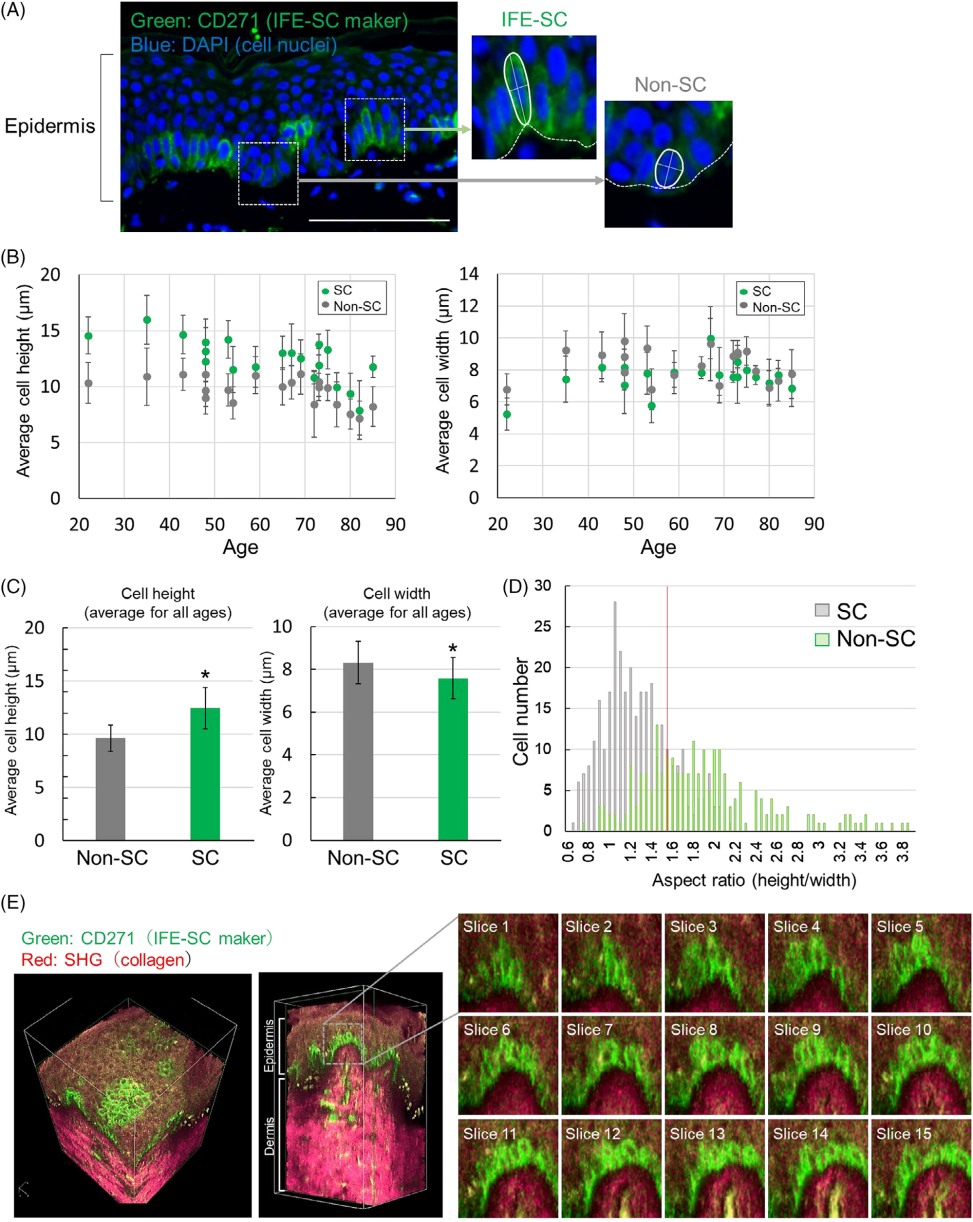
Exploration of the cell morphology characteristics of IFE‐SCs. (A) Immunohistochemical staining of IFE‐SCs in the skin tissue (white bar = 100 µm). Green represents CD271, and blue represents DAPI (4′,6‐diamidino‐2‐phenylindole) (cell nuclei). (B) Quantification of cell morphology (height and width) for both SCs and non‐SCs. (C) Calculation of the mean height and width of SC and non‐SC cells for each specimen (mean ± SD, *N* = 20, *p < 0.05). (D) Histogram illustrating the computed cell aspect ratio (height/width) across all samples analyzed for cell morphology. The red line indicates an aspect ratio of 1.55, used in the discriminant analysis method for classifying IFE‐SCs. (E) Whole‐mount immunohistochemical image of IFE‐SCs (white bar = 50 µm). Green indicates CD271, and red indicates collagen (SHG). The interval of the sliced image is 2 µm.

Initially, our focus was on the cellular morphology of IFE‐SCs, operating on the premise that identifying distinctive cellular morphological characteristics would enable the recognition of IFE‐SCs without using labeling agents. To achieve this, we conducted immunohistochemical analysis on skin tissue sections of 20 specimens (Table S1) using an anti‐CD271 antibody, an IFE‐SC marker (Figure 1A). Subsequently, we measured the average maximum height of the cell perpendicular to the basement membrane as well as the maximum width of the orthogonal cell median for CD271‐positive stem cells (SC) and negative non‐SC in the basal layer for each sample. For cell morphology analysis, immunostained fluorescent images were merged with bright‐field images, and the morphology of each cell was measured from the outlines of stem cells and non‐stem cells in the bright‐field images. The results show that SCs often exhibited taller and narrower characteristics than non‐SCs in numerous specimens (Figure 1B). Averaging the parameters of SCs and non‐SCs across specimens further revealed significant morphological distinctions (Figure 1C). Employing discriminant analysis on the aspect ratio (height/width) of 219 SCs and 314 non‐SCs, we determined that cells with a long spherical shape and an aspect ratio of 1.55 or more could be classified as IFE‐SCs (Figure 1D). Subsequent whole‐mount immunostaining of IFE‐SCs in three dimensions confirmed their localization in the epidermis, displaying a long spherical form consistent with the section analysis (Figure 1E, and Supplementary Video).

**FIGURE 2:**
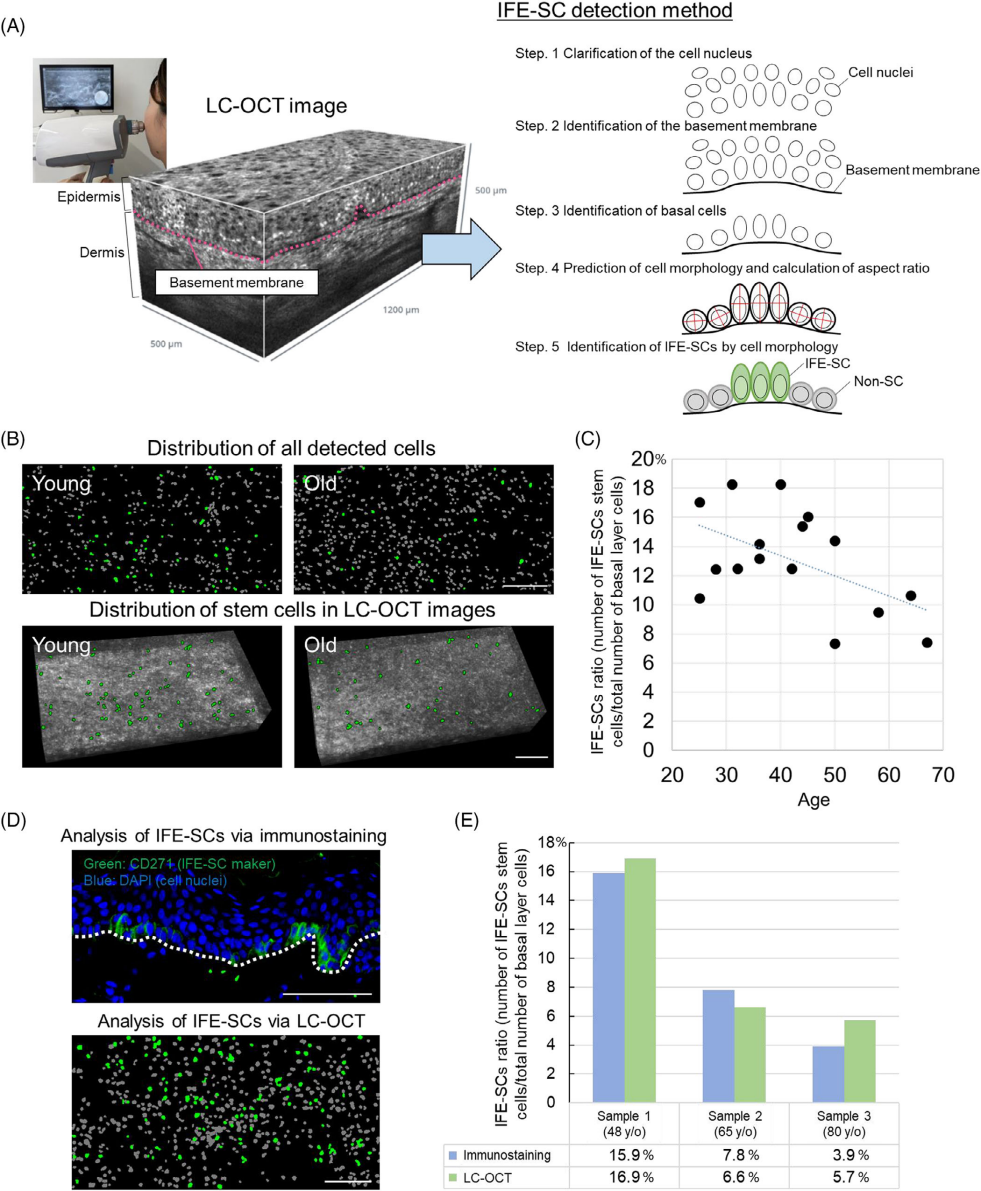
Identification of IFE‐SCs and evaluation of aging‐related changes using LC‐OCT. (A) Utilization of LC‐OCT for obtaining internal structure images of the skin and the IFE‐SC detection process. (B) Exemplary data illustrating IFE‐SCs detected by LC‐OCT in individuals of different ages (young age: 25 years, old age: 67 years) (white bar = 100 µm). Green represents IFE‐SCs, and gray represents non‐SCs. (C) Calculation of the proportion of detected IFE‐SCs relative to the total number of basal layer cells. (D) Comparative image of a 48‐year‐old donor, demonstrating the results of IFE‐SC analysis through immunostaining and LC‐OCT (white bar = 100 µm). (E) Quantitative comparison of IFE‐SCs analyzed through immunostaining and LC‐OCT.

We then hypothesized that conducting in vivo imaging of basal cells within the skin could enable the noninvasive identification of IFE‐SCs based on the cell aspect ratio. A confocal laser biomicroscope, such as the VivaScope, was considered for its ability to noninvasively visualize the internal skin structure. Attempts were made to perform morphological analysis of basal cells using the VivaScope; however, the resolution was insufficient to discern cell morphology (data not shown). In contrast, line‐field confocal optical coherence tomography (LC‐OCT) has recently emerged. LC‐OCT combines the technical principles of optical coherence tomography (OCT), a method utilizing the interferential properties of light to achieve high‐resolution and high‐speed imaging of internal structures, with reflective confocal microscopy employing line‐field illumination. LC‐OCT is capable of generating clearer and more practical 3D images of the skin noninvasively. LC‐OCT images of the buccal epidermis were obtained, revealing numerous epidermal cell nuclei (Figure S1). However, to analyze IFE‐SCs, understanding cell morphology rather than nuclei is essential. Moreover, the detected nuclei morphology was either distorted or more than two nuclei were observed to be joined together. Therefore, machine learning and image processing was employed to segregate individual nuclei and predict cell shape based on nuclear shape. In summary, machine learning was first utilized to segment single nuclei in the acquired LC‐OCT images. Next, the basement membrane was extracted and nuclei proximal to the extracted basement membrane were designated as the nuclei of basal layer cells. Subsequently, cell morphology was predicted based on nuclear morphology. The aspect ratio of the detected basal cells was then calculated, with cells possessing an aspect ratio exceeding 1.55 (Figure 1H) classified as IFE‐SCs (Figure 2A; Supplementary Materials and Methods). LC‐OCT images were acquired from the buccal region of 16 subjects aged 25−67 years (Table S2), enabling the representation of cells inferred as IFE‐SCs in the basal layer (Figure 2B). Additionally, the number of IFE‐SCs was observed to decrease in older skin compared to younger skin (Figure 2B and c). This observation aligns with previous reports indicating a decline in IFE‐SCs with age and is consistent with our study (Figure S2),^3^ validating the detected IFE‐SCs. Furthermore, efforts were made to validate the reliability of our method by comparing the analysis results of IFE‐SCs obtained from LC‐OCT images at the specimen surface with those from conventional immunostaining of the same tissue (Figure 2D). The results demonstrated close proportions of IFE‐SCs in the three analyzed samples (Figure 2E), suggesting high detection sensitivity of IFE‐SCs using this method.

This study has showcased the feasibility of detecting IFE‐SCs through the identification of their morphology, with application to LC‐OCT images. Owing to its noninvasive and label‐free nature, this technology is expected to be efficient for IFE‐SCs analysis and may be useful in evaluating an individual's skin aging status based on the IFE‐SC count as well. However, it is noteworthy that the current analysis involves a substantial processing time and not all cells in the basal layer can be captured. Therefore, we aim to improve the efficiency and accuracy of the analysis, promoting the practical application of IFE‐SCs analysis using this method.

This study received approval from the Ethics Committees of Fujita Health University and Nippon Menard Cosmetic Research Laboratories. Written informed consent was obtained from each subject or their legal guardian before surgery at Fujita Health University Hospital.

## CONFLICT OF INTEREST STATEMENT

The authors state no conflict of interest.

## Supporting information

Supporting information

Supporting information

## Data Availability

Research data are not shared.
