# Supplementary material for: Development of a noninvasive and label‐free imaging system for human interfollicular epidermal stem cells based on cell morphology
Source: Skin Res Technol. 2024 Jul 31;30(8):e13887. doi: 10.1111/srt.13887 (PMC11291864; doi:10.1111/srt.13887)
Supplement: Supplementary file 1 — Supporting information [file SRT-30-e13887-s002.pdf]

Supplementary Materials and Methods

Human skin biopsies

Samples of human skin were collected from surplus surgical tissues from 20 Japanese subjects (Supplementary Table S1) at Fujita Health University Hospital after ensuring that the patients fully understood the study objectives and other related information. Written informed consent was obtained from each subject. This study was conducted upon receiving ethical approval from the Research and Ethics Committee of Fujita Health University (approval No. 15-235).

Clinical photographs

We analyzed the internal structure of living cheek skin from 16 Japanese female subjects (Supplementary Table S1) via line-field confocal optical coherence tomography (LC-OCT, DAMAE Medical, Paris, France). Confocal continuous images of the cheek skin were acquired and three-dimensional processing was performed.

Immunohistochemistry and cell morphology analysis

For whole-mount immunostaining, the skin tissue sample was blocked with 1% BSA for 6 hours and then incubated with a primary antibody against CD271 (Origene, Rockville, USA) for 2 days at 4℃. Thereafter, the samples were incubated with an Alexa Fluor 488-conjugated secondary antibody (Life Technologies, Carlsbad, CA, USA) for 1 day at 4℃. The labeled samples were then immersed in RapiClear 1.52 solution (SunJin Lab, Hsinchu, Taiwan) before being imaged using the two-photon excitation microscope A1RMP (NIKON INSTECH, Tokyo, Japan). Collagen was visualized via second harmonic generation (SHG). For staining CD271 of paraffin-embedded skin sectioned samples, the samples were deparaffinized and boiled in Target Retrieval Solution (Dako, Glostrup, Denmark). After being washed with PBS, these sections were blocked with 2% BSA for 1 hour and then incubated with primary antibodies against CD271 overnight at 4℃. Thereafter, the samples were incubated with Alexa Fluor 488-conjugated secondary antibodies. Cell nuclei were stained with DAPI. Fluorescent images were obtained using the fluorescence microscope BZ-X710 (Keyence, Osaka, Japan). For cell morphology analysis, CD271-positive stem cells and CD271-negative non-stem cells were merged with brightfield images taken simultaneously, and the morphology of each cell was measured from the cell outline visible in the brightfield images using ImageJ (NIH).

Detection of IFE-SCs in LC-OCT images

IFE-SCs were detected using the following procedure:

Step. 1 Using an LC-OCT system (manufactured by DAMAE Medical), 144 internal skin images (width 1072 μm, depth 344 μm) of the cheek were acquired at 1-μm intervals in the depth direction (hereinafter referred to as "LC-OCT images"). Then, to remove noise, 3D Gaussian Blur ($\sigma = 0.8$) was applied to the LC-OCT images using ImageJ. First, cell nuclei were segmented from LC-OCT images. For segmentation, we used the deep learning model StarDist, which was trained on a dataset of segmented LC-OCT images (depth 64 μm, width 128 μm, depth 128 μm). Note that segments with a volume of 150 μm^3 or more and 400 μm^3 or less were considered to be two cell nuclei joined together and were separated using the following procedure. First, principal component analysis was performed on the segments to obtain samples of first principal component scores. For this sample, we determined the separation point at which the degree of separation, which is the ratio of between-class variance and within-class variance, was maximum. When the degree of separation exceeded 3.0, the segment was separated into two by a plane passing through the separation point with the first principal component vector as the normal vector.

Step. 2 Next, the basement membrane was extracted from the detected cell nuclei to select cell nuclei that were close to the basement membrane (hereinafter referred to as "basal cell nuclei"). For extraction, we used a deep learning model U-Net trained with vertical cross-sectional images (height 144 μm, width 1072 μm, 344 images) created from LC-OCT images.

Step. 3 Then, each cell nucleus whose shortest distance to the basement membrane was 4 μm or less was defined as a basal cell nucleus. Furthermore, stem cell nuclei were selected from these basal cell nuclei using the following two procedures. First, they were selected based on the angle formed between the long axis direction of the basal cell nucleus and the basement membrane. When we obtained a sample of the angle between the basal cell and the normal vector of the nearby basement membrane in the section image in advance, the lower 95% point was 45° . Therefore, in LC-OCT images, those in which the angle between the first principal component vector of the basal cell nucleus and the normal vector of the approximate plane of the basement membrane adjacent to the basal cell nucleus was 45° or less were considered candidates for stem cell nuclei.

Step. 4 Next, the abovementioned stem cell nucleus candidates were further selected using the length-to-width ratio of the predicted cell body shape. First, when we calculated the average size ratio of the cell body to the cell nucleus in the section image in advance, it was 1.7 times in the long axis direction and 1.4 times in the short axis direction. Based on this, the length of the long axis of the cell nucleus in the LC-OCT image was determined as the length of the first principal component score, and the length of the short axis was determined as the distance between the two points that are the maximum and minimum of the third principal component score, respectively. The cell body size was predicted to be 1.7 times the long axis of the cell nucleus and 1.4 times the short axis.

Step. 5 Furthermore, when samples of the length-to-length ratio of non-SCs and SCs were obtained in advance from immunostained section images and discriminant analysis was applied, the separation point was 1.55. Based on this, basal cells with a predicted length-to-width ratio of cell bodies exceeding 1.55 were designated as "stem cells." To examine the validity of the above procedure, the stem cell rate was calculated from the numbers of detected stem cells and basal cells, and the correlation coefficient with the age of the subject from whom the LC-OCT image was acquired was calculated. In addition, to compare the results with the stem cell rate obtained using biochemical methods, we obtained immunostained and LC-OCT images of the excised skin section of the same specimen and compared the stem cell rate calculated from both.

Statistical analysis

Data are presented as the mean ± SE. $p < 0.05$ was considered significant. Statistical analysis was performed using Student's t-test.

Supplementary Figure S1

a

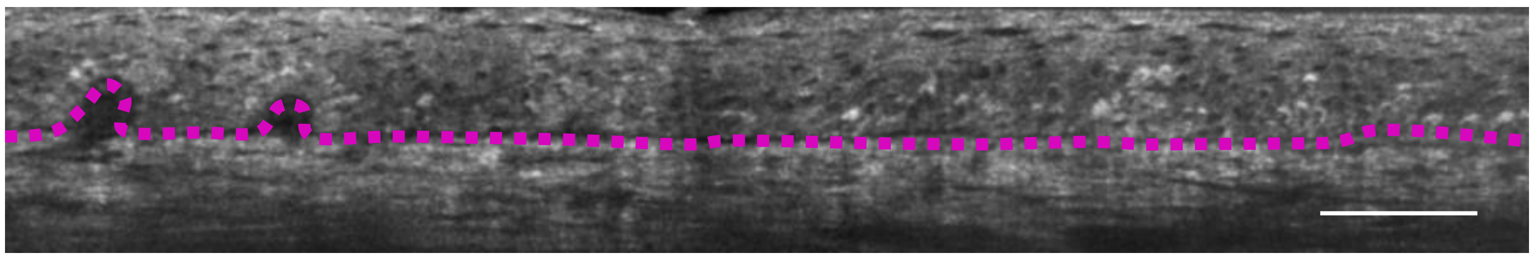

b

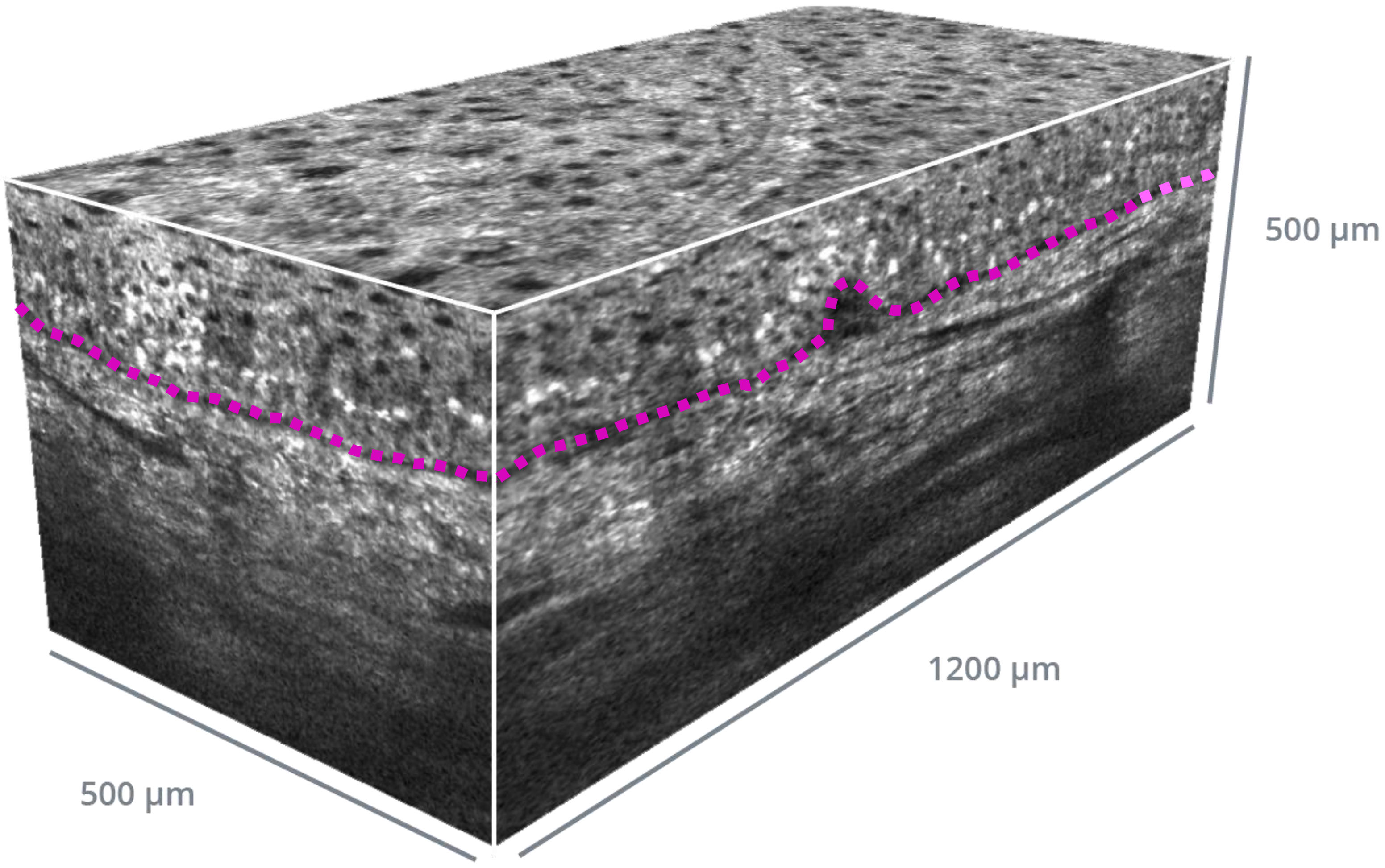

Supplementary Figure 1
(a) The internal structure of the human facial and cheek skin was visualized using LC-OCT. The dotted line indicates the basement membrane (white bar = 100 µm). (b) Three-dimensional reconstructed image of skin internal structure obtained via LC-OCT. The dotted line indicates the basement membrane.

Supplementary Figure S2

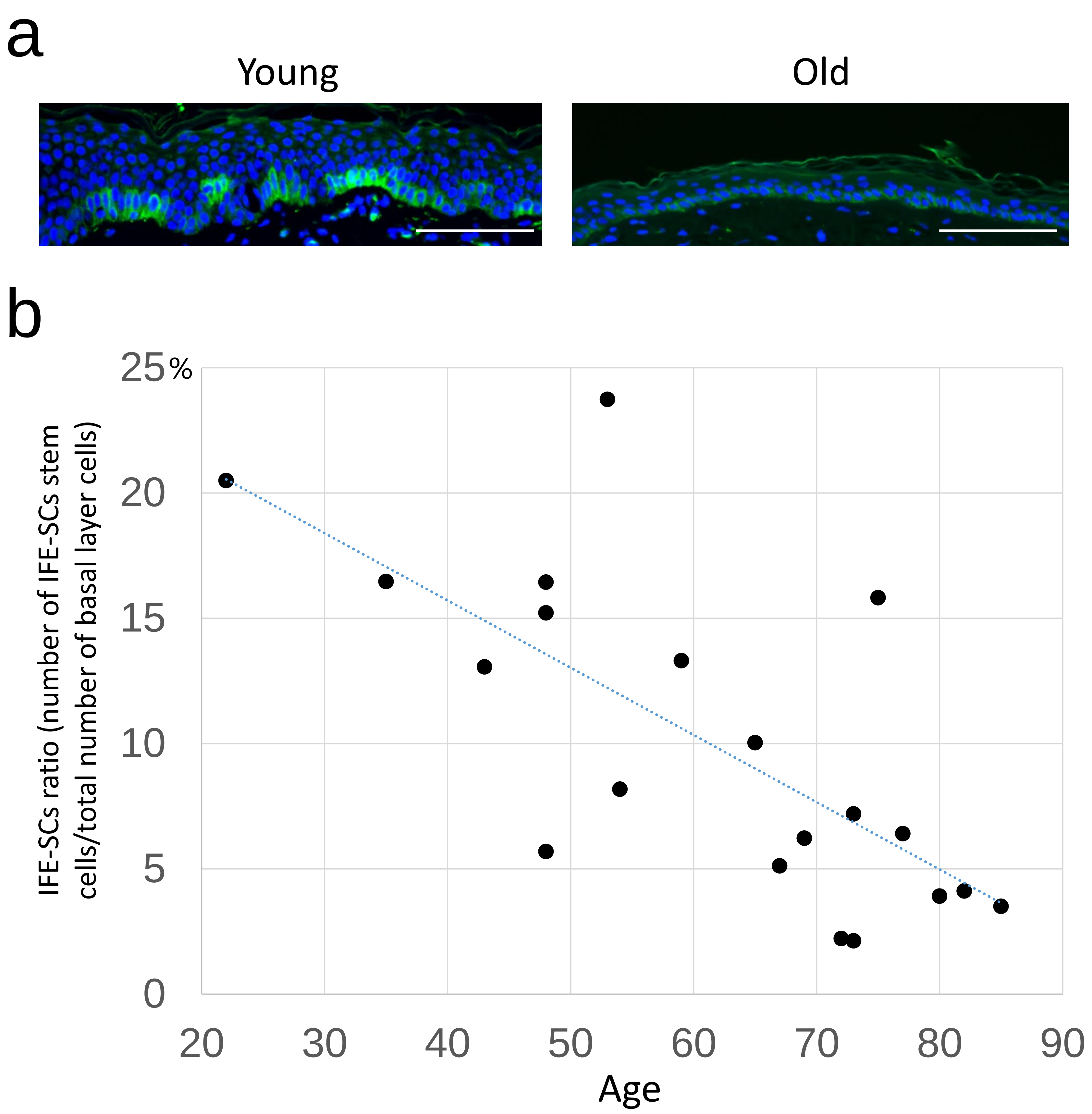

Supplementary Figure 2

(a) Representative image of IFE-SCs in a skin tissue section stained with CD271 antibody. Young: 22 years old, old: 85 years old. (white bar = 100 µm). (b) The ratio of the number of stained IFE-SCs to all cells in the basal layer was calculated.

Supplemental Table S1

Supplementary Table S1. Skin tissue by age and sex of the subjects used for Immunostaining analysis.

Age	Sex	Number of SCs subjected to morphological analysis	Number of non-SCs subjected to morphological analysis
22	M	17	17
35	M	24	24
43	M	15	27
48	M	15	16
48	F	8	16
48	F	16	24
53	M	15	13
54	M	13	15
59	M	11	16
65	M	6	9
67	F	10	23
69	F	9	18
72	F	4	7
73	M	5	6
73	F	6	17
75	M	15	16
77	F	6	10
80	M	13	25
82	M	4	9
85	M	7	6
	**Total**	219	314

Supplemental Table S1

Supplementary Table S2. Skin tissue by age and sex of the subjects used for LC-OCT analysis.

Age	Sex
25	F
25	F
28	F
31	F
32	F
36	F
36	F
40	F
42	F
44	F
45	F
50	F
50	F
58	F
64	F
67	F
